# Double gallbladder with different disease entities: A case report

**DOI:** 10.4103/0972-9941.25673

**Published:** 2006-03

**Authors:** R Vijayaraghavan, Charalingappa S Belagavi

**Affiliations:** Consultant General and Laparoscopic Surgeon, Consultant Pathologist, Raj Mahal Vilas Hospital, 138, AECS Layout, Sanjaynagar, Bangalore - 560094, India

**Keywords:** Double gallbladder, laparoscopic cholecystectomy

## Abstract

We report a rare case of gallbladder duplication in a young male patient with acute pyocoele in one vesicle and acute cholecystitis with cystadenoma in the other; another unusual feature was the absent or obliterated cystic duct in the proximal vesicle and non-communication with the second vesicle or the biliary system. Ultrasound examination had suggested a septate gallbladder; the diagnosis of dual gallbladder was made per-operatively during separation of the distal moiety which was presumed to be an adherent duodenum initially. Intraoperative cholecystogram confirmed the diagnosis and both gallbladders were removed successfully laparoscopically.

A high degree of awareness, detailed preoperative investigations when anomalies are suspected and intraoperative cholangiography are necessary for accurate detailing of the biliary tree to avoid inadvertent damage to the biliary ductal system and overlooking of second or third gallbladder during surgery.

## INTRODUCTION

Gallbladder duplication is an unusual biliary anomaly with a reported incidence of 1:4000 in autopsy studies of humans.[1,2] Lack of awareness, non-specific symptoms and signs and inadequacy of imaging methods are possible reasons for the reported problem of overlooking of the additional gallbladder/s during surgery. An accurate, preferably preoperative diagnosis, identification and removal of all gallbladders during laparoscopy is mandatory to prevent inadvertent damage to the biliary ductal system and possible overlooking of the second or third gallbladder.

## CASE REPORT

**Figure 1:**
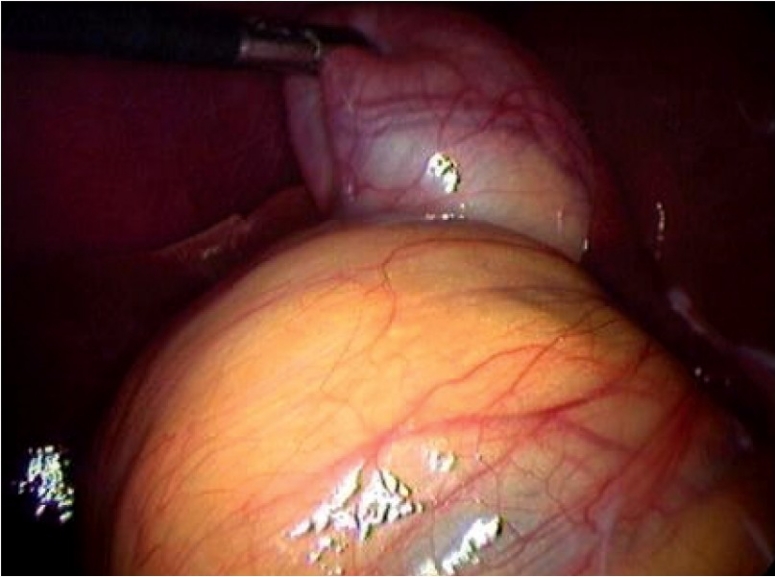
Note the distended lower structure initially mistaken for duodenum

**Figure 2:**
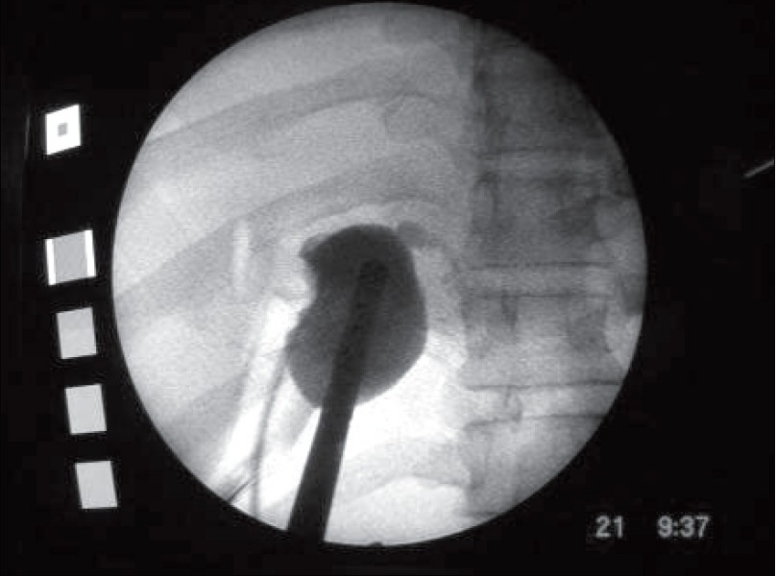
Contrast injection into the lower structure confirms it to be a separate gallbladder with a cystic duct

A 32-year-old male presented to us following an acute attack of severe right upper abdominal pain for two days. Upper abdominal ultrasonography revealed a septate edematous gallbladder with stones; the extra and intrahepatic biliary tree was otherwise normal. Hematological investigations including liver function tests were normal. Laparoscopy using a standard four-port technique showed a more laterally placed thick-walled gallbladder and an edematous cholecystohepatic ligament. [Figure 1] Fundus first dissection of the gallbladder was carried out after aspirating the contents of the gallbladder; initially mucoid content and then frank pus was noted. During dissection at the neck of the gallbladder and in an attempt to separate it from the adjacent structure, bile leak occurred from a small rent in what appeared to be a distended duodenum. Suspecting a duodenal injury, a Fr 8 infant feeding tube was inserted into the rent and contrast injected. A well-distended gallbladder with a normal cystic duct was recognised [Figure 2]. This second gallbladder had no communication with the cephalic gallbladder and was separated from it by edematous connective tissue. The second gallbladder was edematous, inflamed and covered by omental adhesions. A careful search in the liver bed showed no evidence of bile leak or ductal structure and it was assumed that the enlarged cephalic gallbladder either had no cystic duct or the cystic duct was completely obliterated. A separate artery was seen arising from the right hepatic artery and supplying the cephalic gallbladder with the artery coursing in the edematous cholecystohepatic ligament. The distal gallbladder was dissected off the duodenum and after carrying out an intraoperative cholangiogram cholecystectomy was completed. The IOC showed a normal biliary tree and no leakage of dye. Careful inspection of the gallbladder bed showed no biliary leak and no ductal structure could be identified. The patient recovered well and was discharged on the third postoperative day.

**Figure 3:**
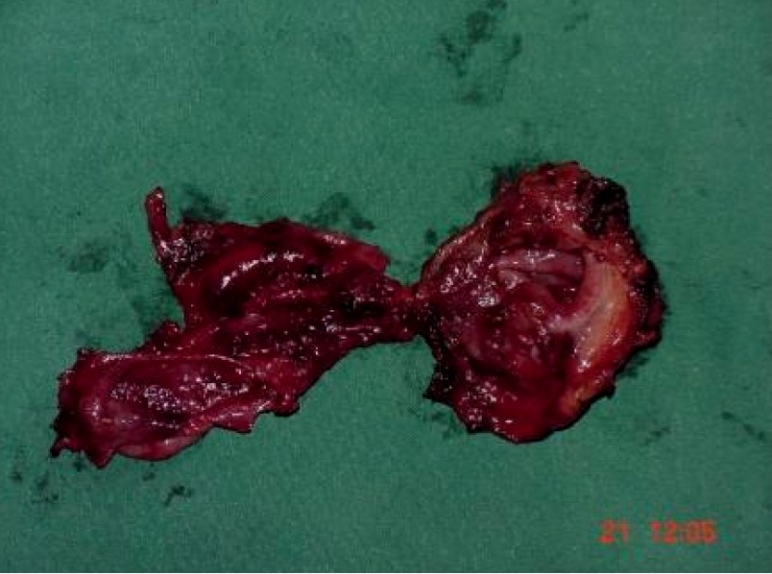
Note the two separate gallbladders

Gross examination of the gallbladders [Figure 3] showed two separate entities with no communication. The cephalic moiety showed features of a pyocoele with severe inflammation of the entire wall. The distal gallbladder showed acute inflammatory changes with cystadenomatous changes. No obvious calculi were seen in either of the gallbladders.

## DISCUSSION

During the fifth or early sixth embryonic week, occasionally, the gallbladder primordium bifurcates and results in duplication of gallbladder. Although estimated to occur once in every 4000 autopsies, the incidence of reported symptomatic cases is probably very low.[1]

Duplication results from a split primordium whilst a true accessory gallbladder results from an extra primordium. It is difficult to tell whether our case represents a true accessory gallbladder or is an actual duplication.

There are no specific symptoms or signs associated with multiple gallbladders; the disease entities reported in the second or occasional third moiety include cholelithiasis, cholecystitis and carcinoma. Occasional case reports of post-cholecystectomy patients having a second attack of cholecystitis have been published.

Ultrasound, MRCP, CT scan, scintigraphy and oral cholecystography have their limitations and are not 100% sensitive in identifying biliary ductal anomalies. The ultrasonographic appearance may be confused with choledochal cysts, gallbladder diverticulum, pericholecystic fluid collections, focal adenomyomatosis, Phrygian cap, extrinsic fibroud bands across the gallbladder and a folded gallbladder Horattas. ERCP may be an useful adjunct but may not be really indicated in every case of Cholelithiasis or Cholecystitis. The surgeon may thus be faced with the prospect of a familiar looking gallbladder and an additional structure that might be missed or presumed to be normal. This could account for the missed cases reported in the literature and in those who underwent a second operation for a diseased remnant gallbladder. Operative cholangiography or cholecystography, in suspected cases should help identify the additional structure.

The anatomical location can vary; most gallbladders share a common peritoneal coat and are usually adjacent to each other. Occasionally, one gallbladder could be entirely intrahepatic or even sub-hepatic. True gallbladder duplications may share a common cystic duct, arterial supply or have separate cystic ducts and blood supply.[1,2,4] In our case, although the arterial supply was separate, no cystic duct could be identified in the cephalic gallbladder. The lack of bile in the cephalic gallbladder, the presence of a pyomucocoele indicates an obliterated cystic duct and non-communication with the biliary tree.

Careful appraisals of reported literature clearly emphasizes the need for removal of accessory or duplicate gallbladders to prevent surgical complications and repeated explorations.[2–8]Asymptomatic gallbladder anomalies are left alone. A search of the English literature identified only 13 reported cases of laparoscopically successfully managed double cholecystectomy. [Table 1]

**Table 1:** Cases of successful laparoscopic management of double gallbladders reported in the English literature

Author and year reported	Age and gender of patient	Preoperative diagnosis	Intraoperative diagnosis	Intraoperative difficulty	Disease process
Garcia J, 1993	21 yr Male	-	Yes	No, but only partial cholecystectomy	Cholelithiasis in both
Miyajima N et al, 1995	28 yr Male	Yes	-	No	Cholelithiasis in both
Cummiskey et al, 1997	39 yr Female	-	Yes	No	Cholelithiasis no changes
Gigot et al, 1997	29 yr Female	Yes	-	Difficult dissection; staged removal	Cholelithiasis recurrent cholecystitis
Horattas MC, 1998	35 yr Female	Yes	-	No	Chronic cholecystitis in both
Maddox, 1999	**	**	**	No	**
Tsutsumi et al, 2000	74 yr Female	Yes	-	No	Cholelithiasis in both
Kaya Yorganci et al, 2001	48 yr Female	-	Yes	No	Cholecystitis no changes
Vahit Ozmen, 2003	34 yr Female	Yes	-	No	Chronic inflammation erosions
Yasuo O et al, 2003	44 yr Male	Yes	-	No	Cholelithiasis adenomyomatosis
		Yes	-	No	Cholesterolosis in both
Amit Goel et al, 2003	25 yr Female	Yes	-	No	Cholelithiasis
Shirahane et al, 2003	61 yr Female			
Claudio C M et al, 2004	36 yr Female	-	Yes	No	Cholelithiasis no changes
Present case, 2005	32 yr Male	-	Yes	No	Pyocoele acute cholecystitis and cystadenoma

** Details not available

Congenital anomalies of the gallbladder are rare and may cause clinical, surgical and diagnostic problems. If not preoperatively diagnosed, overlooking of the additional gallbladder during surgery may result in recurrence of symptoms or biliary complications. Intra-operative cholecystography and cholangiography is essential to clarify the ductal anatomy and to help identify additional anomalous structures.

Meticulous dissection of any additional cystic or tubular structures in the subhepatic region, identification of anatomical details prior to transection along with a high degree of awareness of gallbladder anomalies could possibly prevent catastrophic consequences. Previously, there has been stress on the need for open procedures to identify these anomalies. In the present scenario, with expertise, these cases could be tackled by laparoscopy successfully and with minimal morbidity. Laparoscopic magnification offers a clear picture of details and a skilled laparoscopic surgeon may not need to convert these cases to open procedures. Standard 4-port technique is generally sufficient; occasionally, an additional port is needed to facilitate retraction of adjacent structures. Intraoperative cholangiography is helpful in clarification of ductal details and is recommended in all such cases.

## CONCLUSION

Gallbladder anomalies are rare entities and may be a trap for the unwary surgeon. Laparoscopic management is a feasible option with minimal morbidity. Intraoperative delineation of the biliary tree by contrast injection outlines the possible anomalies facilitating meticulous dissection and complete removal of all gallbladders.
